# Clinical pregnancy in Turner syndrome following re-implantation of cryopreserved ovarian cortex

**DOI:** 10.1007/s10815-023-02905-w

**Published:** 2023-08-11

**Authors:** CE Dunlop, SA Jack, EE Telfer, S. Zahra, RA Anderson

**Affiliations:** 1 Simpson’s Centre for Reproductive Health, Edinburgh Royal Infirmary, Edinburgh, EH16 4SA UK; 2 Institute of Cell Biology and CDBS, University of Edinburgh, Edinburgh, EH8 9XD UK; 3 Tissues, Cells & Advanced Therapeutics, Scottish National Blood Transfusion Service, Edinburgh, EH14 4BE UK; 4 MRC Centre for Reproductive Health, University of Edinburgh, Edinburgh, EH16 4TJ UK

**Keywords:** Turner syndrome, Ovarian cortex re-implantation, Fertility preservation, Premature ovarian insufficiency

## Abstract

Turner syndrome (TS) leads to a characteristic phenotype, including premature ovarian insufficiency and infertility. Ovarian tissue cryopreservation (OTC) is becoming an established fertility preservation strategy for both pre- and post-pubertal females and may offer the chance of having a biological family to selected patients with TS. To date, women with TS have had ovarian tissue cryopreserved but there are few reports of autologous re-implantation and none of pregnancy. We herein report, to our knowledge, the first clinical pregnancy in a patient with TS, conceived naturally following re-implantation of cryopreserved ovarian tissue which had been removed soon after spontaneous puberty. This provides proof of concept for OTC as a means of fertility preservation in TS.

## Introduction

Turner syndrome (TS) is a rare condition affecting approximately 50 in 100,000 women [1]. Its characteristic phenotype includes short stature, delayed puberty, hypergonadotrophic hypogonadism, infertility and an increased risk of cardiovascular, endocrine and autoimmune disease. The syndrome is caused by a deficiency of X-chromosome material, with almost half of women with TS possessing a 45,XO karyotype. Other karyotypes include mosaicism (e.g. 45,XO/46,XX) and the presence of an isochromosome of the Xp or Xq arm, Y-chromosomal material or ring chromosome (where the X-chromosome fuses at its ends to form a ring structure, thus reducing its genetic content) [2].

The long arm of the X-chromosome appears to be integral to fertility [3]. In TS, ovaries contain significantly fewer follicles due to widespread accelerated oocyte apoptosis [4, 5]. Only a third of patients spontaneously enter puberty and only 15–20% experience menarche [6–8], with premature ovarian insufficiency (POI) usually occurring at a young age. Natural conception is therefore uncommon: a study of almost 500 women demonstrated a spontaneous pregnancy rate of only 5.6% and a doubling of the miscarriage rate of that of the general population [6].

The appropriateness of offering fertility preservation in those with TS has been debated [9, 10]. Although oocyte cryopreservation is possible, it is only feasible in the proportion of those with TS who are post-pubertal and these adolescents and women are more likely to have 45,XO/46,XX mosaicism as this is associated with a less severe phenotype [11, 12].

Ovarian tissue cryopreservation (OTC) for fertility preservation can be performed both pre- and post-pubertally and is therefore an option for those with TS, although it remains experimental in that context [10, 13], with very limited data on the re-implantation of cryopreserved ovarian tissue in this cohort. Although there is a case report of a patient with TS mosaicism and POI having a successful pregnancy following allografting of ovarian tissue from the patient’s monozygotic twin who still had ovarian function despite also having TS mosaicism [14], there are no previously reported pregnancies or livebirths in patients with TS undergoing autotransplantation of ovarian tissue. We here describe, to our knowledge, the first clinical pregnancy following re-implantation of cryopreserved ovarian cortex in a woman with TS.

## Case story

### Clinical history

**Fig. 1:**
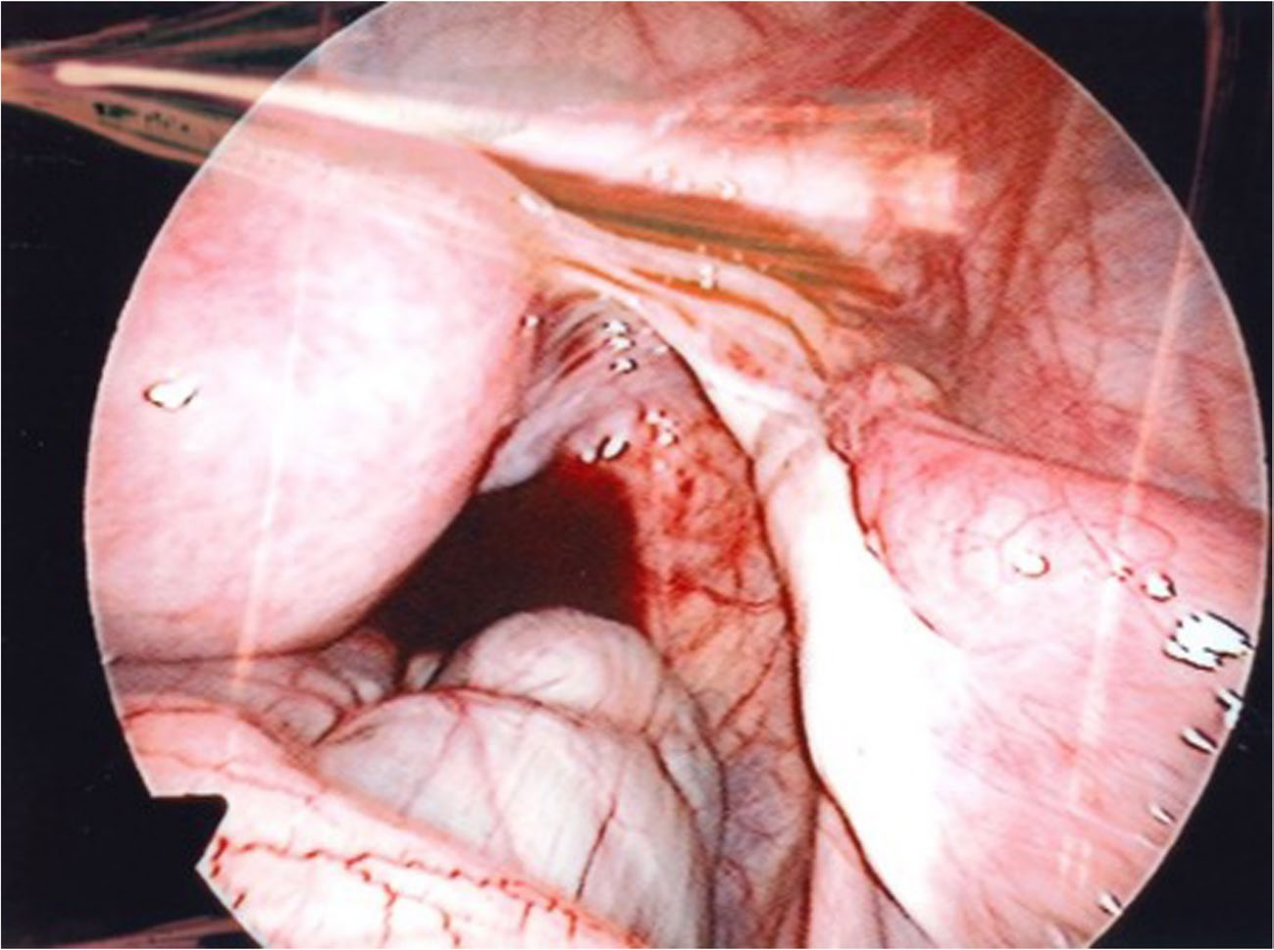
Image from the laparoscopic ovarian cortex biopsy procedure demonstrating a normal uterus, normal right Fallopian tube and small right ovary

The patient was diagnosed with TS mosaicism (45,XO/46,X,ring(X)) in childhood. She received growth hormone treatment and spontaneously entered puberty at 12 years old, with menses from 14 years of age. She had normal echocardiograms and was otherwise fit and well. At the age of 14 years old, her menses were regular and gonadotrophin levels were low (follicle-stimulating hormone (FSH) 1.9 U/L, luteinising hormone (LH) 1.3 U/L) but anti-Mullerian hormone (AMH) concentration was undetectable (<4 pmol/L; subsequently measured at 0.30 pmol/l using a more sensitive assay). She was referred to the Fertility Preservation service in Edinburgh for discussion of OTC. A transabdominal ultrasound at this time demonstrated a normal uterus and normal right ovary (volume 4.6cm^3^), whilst the left ovary was not visualised. The patient and her parent were counselled regarding OTC, highlighting the uncertainties of success, and wished to proceed. At 15 years of age, the patient underwent laparoscopic removal of ovarian cortex biopsies from both ovaries with informed consent and ethics committee approval. Two small ovaries were seen intra-operatively, with otherwise normal pelvic anatomy (Fig. 1).

**Fig. 2:**
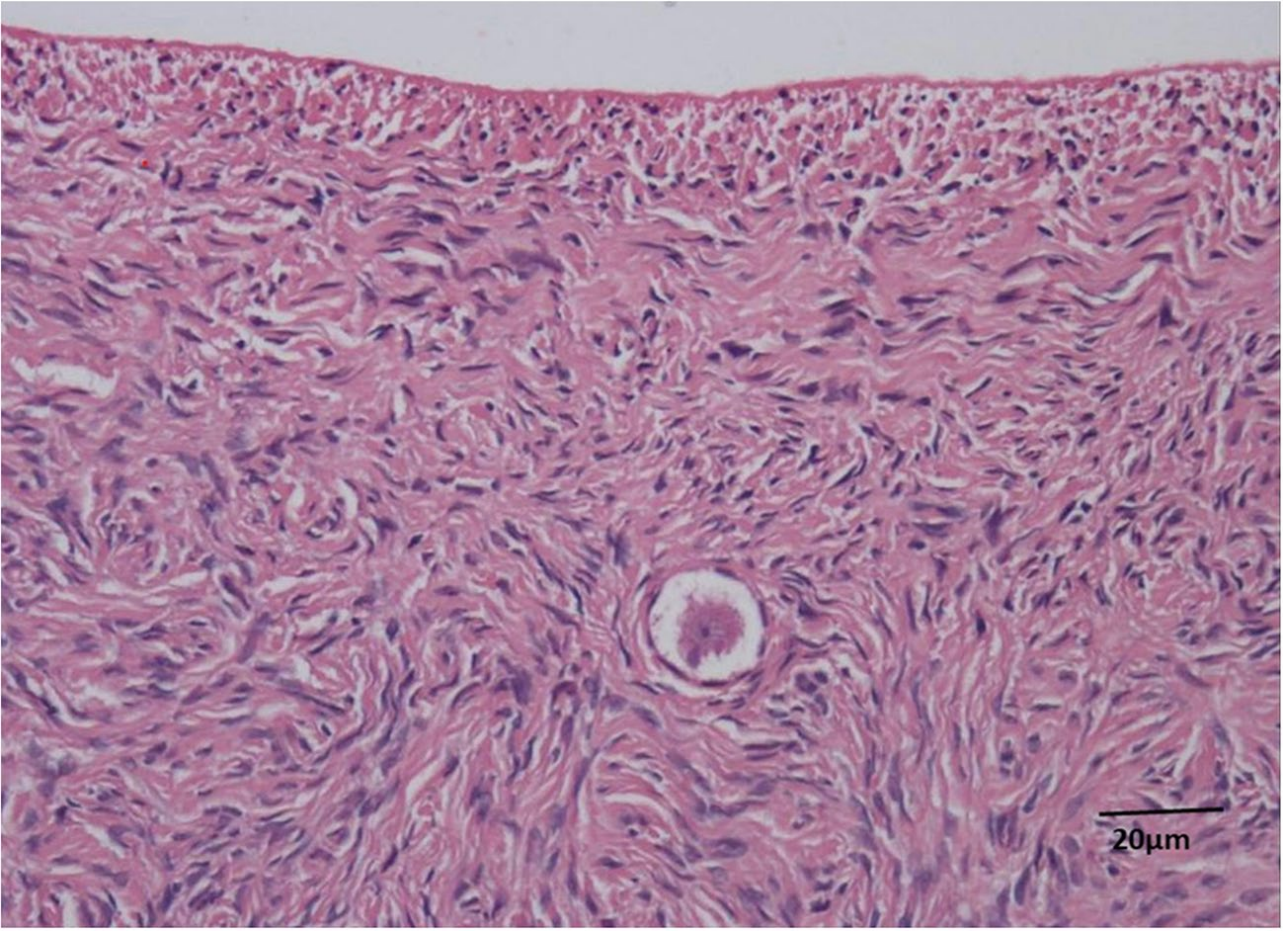
Histological image of a biopsy of ovarian cortex removed during ovarian cortex cryopreservation procedure demonstrating a single primordial follicle, surrounded by normal ovarian stroma

The biopsies were slow frozen [15] and stored in the vapour phase of liquid nitrogen. A sample of ovarian cortex was sent for histopathological analysis (Fig. 2) and follicle density analysis [16] identified 3 non-growing follicles/mm^3^ of ovarian cortex. Subsequent examination of over 140 5 μm sections of tissue showed only empty follicle-like structures.

### Ovarian tissue re-implantation

The patient subsequently transitioned to care under the Adult Turner Syndrome clinic where she was treated for heavy menstrual bleeding, requiring iron supplementation. She was re-referred to the Edinburgh Fertility Centre at 23 years of age as she was in a long-term relationship, was nulliparous and had been trying to conceive for 2 years. She had irregular menses and a raised FSH (14.9 U/L). A transvaginal ultrasound demonstrated an antral follicle count of 3. She was diagnosed with incipient premature ovarian insufficiency (POI) and elected for re-implantation of her cryopreserved ovarian tissue. She had a normal body mass index and was a non-smoker. Her male partner had unproven fertility, and a normal semen analysis.

A laparoscopic orthotopic transplantation of thawed ovarian tissue was performed when the patient was 24 years old. At operation, the right ovary contained a small (approx. 1.5cm) cyst, whilst the left ovary was small. The uterus and fallopian tubes were normal. Ten pieces of ovarian cortex were thawed, and inserted into both ovaries, including into the drained cystic structure.

### Clinical pregnancy

Due to the COVID-19 pandemic, systematic follow-up of the patient was not possible, although pre-pregnancy counselling was performed approximately 3 months post-re-implantation. The patient reported resumption of regular menstrual cycles 2 months post-operatively, for 4 months, before becoming more erratic again. She was reviewed 8 months post-re-implantation when she was on day 16 of her cycle and a transvaginal ultrasound demonstrated a pre-ovulatory follicle (20.7mm in diameter) on the right ovary, with a thickened endometrium of 11.5mm. Her estradiol level was 1453 pmol/L and an hCG trigger injection was administered. She subsequently had a positive urinary pregnancy test and an ultrasound at 6^+0^ weeks gestation demonstrated a single intrauterine gestational sac, a yolk sac and a fetal pole with fetal heart pulsations. The crown-rump length (CRL) was 3.8mm. Unfortunately, she experienced per vaginum bleeding a few days later and a repeat ultrasound showed appearances consistent with a missed miscarriage, with no increase in the CRL, no fetal heart pulsations and a haematoma within the uterine cavity. She underwent uncomplicated medical management of miscarriage: products of conception were not collected for analysis.

She had two more cycles monitored by ultrasound scanning and blood tests, which both showed an ovulatory follicle, estradiol level >1000 pmol/L, and progesterone level measured in one cycle at 64.3 nmol/L following hCG trigger injections. She subsequently withdrew from active management in the fertility setting but continued at most recent review to have regular menstrual cycles 2 years after tissue replacement.

## Discussion

In this case report, we describe the first clinical pregnancy in a woman with TS who had undergone re-implantation of autologous ovarian cortex cryopreserved shortly after spontaneous puberty. The tissue had been cryopreserved for almost 9 years. Regular menses resumed shortly after re-implantation and the clinical pregnancy was naturally conceived, although sadly did not result in a livebirth. Although OTC has been offered to young girls and women with TS for many years, there are limited data regarding the success of ovarian tissue re-implantation in this cohort. In a study of 100 patients with TS undergoing fertility preservation counselling over a period of 22 years, 73 patients opted for OTC, but only 62 had biopsies taken. Only 2 (3.2%) patients returned for re-implantation and neither of them regained endocrine function post-operatively [17]. A separate cohort study in the Netherlands aiming to recruit 100 patients with TS undergoing OTC is underway [18]. The paucity of data is reflected in an international consensus guideline for the care of TS, which, although recognising OTC as a feasible fertility preservation option, did not include it within its recommendations [2], and the more recent ESHRE guideline recommends that it is performed only within a research protocol [10], as here.

The feasibility of OTC in TS is backed up by follicular data, with normal follicles seen on histological analysis of ovarian cortex biopsies of some women with TS [16, 19]. The presence of follicles is more likely in those who have undergone spontaneous puberty, have mosaicism and normal FSH and/or AMH levels. However, a high proportion of these follicles may be abnormal and therefore it should only be considered in selected patients; for example, those with the above prognostic indicators, with no evidence of POI and no contraindications to pregnancy [16]. This is exemplified by the present case, who conceived despite a large proportion of abnormal follicles. Analysis of the genetic content of the oocytes within follicles in TS showed that 91% contained an X chromosome, despite the majority of ovarian supporting cells being 45,XO, although the sample size was small [20, 21]: a previous case report provided comparable data [21] Thus it appears that the level of mosaicism in both the oocyte and the ovarian stromal and granulosa cells is not always closely predicted by the peripheral cells typically used for a genetic diagnosis (e.g. lymphocytes and buccal cells) and therefore some, apparently monosomic, TS patients may be in fact have a “hidden” mosaicism and thus be appropriate for consideration of fertility preservation [22]. Nevertheless, such patients are much less likely to have any follicles on ovarian biopsy [16].

Within the wider context of OTC, the largest review of ovarian tissue re-implantation to date, including 285 women from five centres in Europe, demonstrated resumption of menses in 88.7% of those with POI within an average of 4.5 ± 2.2 months, and a natural conception rate of 40% [23]. Although the patient in this case report was not amenorrhoeic at the time of re-implantation, she did experience resumption of regular menstrual cycles within a similar time-frame and achieved natural conception. Our findings also confirm those of previous reports which demonstrate that long-term storage of cryopreserved ovarian tissue is successful, with pregnancies achieved following re-implantation of tissue stored for 10 [24] and 11 years [25].

Sadly, in this case, the pregnancy ended with a first trimester miscarriage. It is well recognised that women with TS experience a higher miscarriage rate than the general population, which may largely be due to resultant abnormal fetal karyotypes [6, 26]. Furthermore, in those with TS who had a livebirth, there is evidence of an increased risk of prematurity and low birth weight [27, 28] and a possible increased incidence of fetal abnormalities [29]. There is also a higher rate of delivery by Caesarean section, due to cephalopelvic disproportion or the cardiovascular risks associated with labour in this cohort of women [30]. Pregnancy-associated hypertensive disorders are also more common: a study of TS patients who received donor oocytes demonstrated that over a third (37.8%) experienced pregnancy-associated hypertension, with over half of these diagnosed with pre-eclampsia [28]. The maternal mortality rate was 2.2%, in both cases due to aortic rupture.

The majority of women having ovarian tissue re-implantation have established POI, although almost a fifth of patients (18.8%) had irregular cycles at the time of re-implantation, albeit with evidence of infertility [23]. This is the case with the patient discussed here: she was experiencing erratic menses with an elevated FSH level and had been trying to conceive for over 3 years. We cannot be certain that the subsequent pregnancy originated from a follicle within the grafted tissue. However, it is clear that she regained regular menses following the re-implantation, and given the duration of her preceding infertility, it would seem entirely plausible that the re-implanted tissue was the source of the fertilised oocyte. The lifespan of the re-implanted tissue is unknown as it was ongoing at most recent review. Data suggests a 5-year graft survival rate of 55% [23]; it would be valuable to ascertain the longevity of such grafts in patients with TS.

There are ethical considerations surrounding the provision of fertility preservation in patients with TS: re-implantation of cryopreserved ovarian tissue has never before been proven to be successful out with the oncological population or those needing gonadotoxic treatment for benign conditions, thus in women without ovarian-based pathology. Given the lack of evidence of its success in those with congenitally abnormal ovaries, such as in TS and galactosaemia [31], it has been posited that the risks of fertility preservation, both physical and psychosocial, may not outweigh the benefits if the procedure does not lead to a livebirth [2, 32]. Indeed, in this case the patient withdrew from active fertility management after her miscarriage. Furthermore, there is risk of precipitating POI in this cohort of patients by the very act of removing ovarian tissue during OTC, and very young patients are being asked to make decisions that they may not be emotionally or psychologically ready for. A recent study of patients with TS and their parents analysed the decision-making process regarding OTC [13]. It demonstrated that the option of OTC provided hope of future fertility and that the requirement for surgery was not off-putting for the majority of patients. However, the paucity of long-term data on success rates was an important consideration in those patients and parents who declined the procedure. This case report demonstrates that OTC and ovarian tissue re-implantation is a feasible fertility preservation option for selected patients with TS, providing evidence that clinical pregnancy can be achieved using this method, thus it may be of value in those undertaking counselling of patients with TS.
